# G-CODE: enabling systems medicine through innovative informatics

**DOI:** 10.1186/gb-2011-12-s1-p38

**Published:** 2011-09-19

**Authors:** Subha Madhavan, Yuriy Gusev, Michael A Harris, David M Tanenbaum, Robinder Gauba, Krithika Bhuvaneshwar, Andrew Shinohara, Kevin Rosso, Lavinia A Carabet, Lei Song, Rebecca B Riggins, Sivanesan Dakshanamurthy, Yue Wang, Stephen W Byers, Robert Clarke, Louis M Weiner

**Affiliations:** 1 Lombardi Comprehensive Cancer Center, Georgetown University Medical Center, 2115 Wisconsin Avenue NW, Suite 110, Washington DC 20007, USA; 2 ESAC, 155 Gibbs Street, Suite 420, Rockville, MD 20850, USA; 3 Bradley Department of Electrical & Computer Engineering, Virginia Tech Research Center - Arlington, 900 North Glebe Road, Arlington, VA 22203, USA

## 

The new and emerging field of systems medicine, an application of systems biology approaches to biomedical problems in the clinical setting, leverages complex computational tools and high dimensional data to derive personalized assessments of disease risk. Systems medicine offers the potential for more effective individualized diagnosis, prognosis and treatment options. The Georgetown Clinical & Omics Development Engine (G-CODE) is a generic and flexible web-based platform that serves to allow basic, translational and clinical research activities by integrating patient characteristics and clinical outcome data with a variety of high-throughput research data in a unified environment to enable systems medicine. Through this modular, extensible and flexible infrastructure, we can quickly and easily assemble new translational web applications with both analytic and generic administrative features. New analytic functionalities specific to the needs of a particular disease community can easily be added within this modular architecture. With G-CODE, we hope to help enable the creation of new disease-centric portals, as well as the widespread use of biomedical informatics tools by basic, clinical and translational researchers, through providing powerful analytic tools and capabilities within easy-to-use interfaces that can be customized to the needs of each research community. This infrastructure was first deployed in the form of the Georgetown Database of Cancer (G-DOC) [1], which includes a broad collection of bioinformatics and systems biology tools for analysis and visualization of four major omics types: DNA, mRNA, microRNA and metabolites. Although several rich data repositories for high dimensional research data exist in the public domain, most focus on a single data type and do not support integration across multiple technologies. G-DOC contains data for more than 2,500 patients with breast cancer and almost 800 patients with gastrointestinal cancer, all of which are handled in a manner that allows maximum integration. We believe that G-DOC will help facilitate systems medicine by allowing easy identification of trends and patterns in integrated datasets and will hence facilitate the use of better targeted therapies for cancer.

One obvious area for expansion of the G-CODE/G-DOC platform infrastructure is to support next-generation sequencing (NGS), which is a highly enabling and transformative emerging technology for the biomedical sciences. Nonetheless, effective utilization of these data is impeded by the substantial handling, manipulation and analysis requirements that are entailed. We have concluded that cloud computing is well positioned to fill these gaps, as this type of infrastructure permits rapid scaling with low input costs. As such, the Georgetown University team is exploring the use of the Amazon EC2 cloud and the Galaxy platform to process whole exome, whole genome, RNA-Seq and chromatin immunoprecipitation (ChIP)-Seq NGS data. The processed NGS data will be integrated into G-DOC to ensure that they can be analyzed in the full context of other omics data. Likewise, all G-CODE projects will simultaneously benefit from these advances in NGS data handling. Through technology re-use, the G-CODE infrastructure will accelerate progress in a variety of ongoing programs that are in need of integrative multi-omics analysis and will advance our opportunities to practice effective systems medicine in the near future.
